# The influence of the pharmaceutical industry on the development of gonadotrophins and ovarian stimulation protocols in assisted reproductive technologies

**DOI:** 10.3389/fendo.2025.1536844

**Published:** 2025-04-04

**Authors:** Tim Child, Robert Bassett, Colin M. Howles

**Affiliations:** ^1^ Nuffield Department of Women’s and Reproductive Health, University of Oxford, Oxford, United Kingdom; ^2^ Oasthouse Consulting Sàrl, Commugny, Switzerland; ^3^ ARIES Consulting Sàrl, Geneva, Switzerland; ^4^ Honorary Fellow, Deanery of Biomedical Sciences, University of Edinburgh, Edinburgh, United Kingdom

**Keywords:** ovarian stimulation, assisted reproductive techniques, IVF, recombinant FSH, urinary FSH, hMG, gonadotrophins, pharmaceutical industry

## Abstract

**Introduction:**

This review examines the evolution of gonadotrophins in ovarian stimulation (OS) protocols for assisted reproductive techniques (ART). Since the advent of *in vitro* fertilisation (IVF) in the late 1970s, the pharmaceutical industry has played a pivotal role in advancing gonadotrophin production, improving drug purity and optimising delivery methods. Despite significant progress, questions remain about the robustness of the evidence supporting the use of different gonadotrophins and the impact of industry-driven research on clinical practice. The review critically examines the evolution, evidence and future directions of gonadotrophin use in ART.

**Methods:**

A comprehensive literature search was carried out in multiple databases to select articles/reviews on historical developments, manufacturing and analytical techniques, regulatory frameworks and clinical trials undertaken to assess gonadotrophin production, formulation processes and their integration into clinical practice. The analysis included mainly evidence from pharmaceutical sponsored randomised controlled trials (RCTs) as well as single arm, registration or post approval studies. Studies on new molecular entities were reviewed. Systematic reviews and meta-analyses, national registries were consulted. Laboratory developments, regulatory challenges, economic constraints, were considered.

**Results:**

Over the past four decades, ART has seen remarkable improvements, including increased live birth rates in women of advanced ovarian age, reduced multiple births, and the advent of patient-friendly pen devices. Innovations such as recombinant FSH (rFSH) and biosimilars have expanded treatment options. However, the high cost of drug development as well as the complexity of the ART process have contributed to underpowered trials and reliance on meta-analyses, which often fail to account for confounding factors.

**Discussion:**

While gonadotrophins have been shown to be effective for OS, unresolved issues, such as the role of supplementing LH activity in OS protocols, highlight the need for more robust trials. Collaboration between stakeholders is essential to standardise trial designs, define key outcomes and minimise bias. Emerging technologies, including AI and genetic testing, offer opportunities to refine embryo assessment and implantation outcomes, thus improving trial design. A renewed focus on rigorous, transparent trials and interdisciplinary collaboration is essential to advance patient care and address unmet challenges in ART treatment. Beyond gonadotrophins, alternative therapeutic avenues to improve oocyte competence and implantation success warrant exploration.

## 1 Introduction

**Figure 1:**
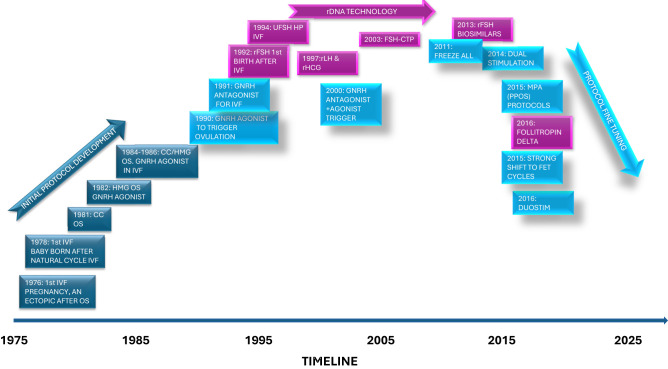
Schematic of some of the important timepoints when ovarian stimulation drugs and protocols were described in the literature.

Before 1985, the field of *in vitro* fertilization (IVF) was beautifully simple, limited information and choice of drugs for ovarian stimulation (OS) and luteal phase support, coupled with off the shelf laboratory equipment and home cooked recipes for the laboratory reagents. Information was readily exchanged between clinics, and pharmaceutical companies were themselves learning about the needs and nature of this exciting new venture. From a very early stage though, advances and refinements in OS regimens (**Figure 1**) and laboratory practices (1) were implemented not only by pharmaceutical or laboratory companies, but also inquisitive and brave individuals who were driven by a desire to improve outcomes, reduce and standardize the complexity of the IVF treatment process.

Forty six years on from the birth of the first IVF baby, Louise Brown, conceived following oocyte recovery in a natural cycle, our field of research and patient care continues to evolve at a tremendous pace; new drug therapeutics, laboratory procedures and innovative devices have been introduced and treatment is shifting from a single isolated intervention, covering approximately four weeks to a longitudinal segmented approach (2) that may occur over potentially many years, hence providing a viable option for individuals undergoing cancer therapy (3), as well as those women who desire to preserve their fertility.

Longitudinal data on live birth rates from the HFEA, the UK fertility regulator, clearly demonstrates the advancements in ART that have been made over the last 40 years especially for women up to the age of 39 as well as a significant reduction in multiple births from a high of 30% to around 4% in 2022 (4 website).

As for overall drug development, it must be appreciated that developing and bringing to market new medicines is high risk and costly. In 2023, it was estimated that European research-based pharmaceutical companies invested an estimated € 50 billion in R&D and directly employs 900,000 people (5). There are increasing challenges, escalating R&D costs and increased regulatory hurdles. From the development of a new active substance to it reaching the market takes around 12-13 years and an average of 1-2 out of 10,000 new substances successfully pass all stages of development and are approved. In the fertility therapeutic area, the most recently launched follitropin product, took around 8 years to reach the market (**Table 1**). Additionally, the whole European sector has been severely hit by the impact of austerity measures introduced by European governments since 2010 (5).

**Table 1:** Examples of drug development time* in the reproductive medicine therapy area.

Molecule	Patent Filed	First Scientific Publication	Clinical Development Time (Phase I-III)	First Regulatory Registration	Drug Development Time (Patent-Registration)
rFSH (follitropin alfa)	1985	1989	1992-1995 (4 yrs)	1995 (Europe)	11yrs
rFSH (biosimilar)	Patent for follitropin alfa expired 2012	2015	2008-2011 (4yrs)	2013 (Europe)	6yrs+
rFSH (long-acting fusion CTP)	1989	1992	2001-2008 (8 yrs)	2010 (Europe)	22yrs
rFSH (follitropin delta)	2009	2014	2014-2016 (3 yrs)	2016 (Europe)	8yrs
GnRH analogue (antagonist- cetrorelix)	1980	1986	1991-1997 (7yrs)	1999 (Europe)	20yrs
GnRH Antagonist (orally active small molecule)	1987	1989	2010-2017 (8yrs)	2018 (USA)	32yrs

*Calculated from the time of patent filing date.

+Calculated from commencement of clinical development.

The decision for a company to undertake any drug, or device development is highly complex (6). For instance, it involves assessing multiple factors, including the commercial opportunity, the level of internal company competition for R&D budget in other therapeutic developments, the patent landscape and/or patent protection, the level of external competition, regulatory requirements as well as the potential burden of proof to achieve the desired selling price, and reimbursement or insurance coverage. Finally, the potential clinical interest in the medical community and the willingness/interest of patients to demand the new treatment options also must be considered. Alternatively, it is less complex and less costly, for pharmaceutical companies to sponsor investigator-initiated studies to either encourage innovation in clinical practice or just to build customer/brand relationships.

Thus, it is a fine balancing act for a pharmaceutical company to decide between maintaining a drug development pipeline through to regulatory approval, as well as investment in post marketing clinical activities, including scientific journal contributions. This has led in our therapeutic area to a plethora of small and medium sized underpowered clinical trials that are then subsequently incorporated into meta-analyses covering ovarian stimulation treatment options (e.g. 7).

For ovarian stimulation, how can we effectively keep pace with our fast-evolving medical discipline; is the body of evidence robust and communicated with full transparency e.g. are there differences between FSH preparations? What lessons can we learn from our relatively short past? In this commentary we will review in detail, the evolution of the manufacturing, formulation and analytical processes used in gonadotrophin production as well as discuss novel development pathways, some of which were adopted by pharmaceutical companies, leading to new market entrants. Finally, we will critically examine the scientific and clinical evidence for gonadotrophin use, as well as propose the future focus for gonadotrophin and ART research and development.

## 2 Pharmaceutical developments in manufacturing

### 2.1 Manufacturing processes

The initial manufacturing and testing of animal and human pituitary gonadotrophins, as well as pregnant mare serum gonadotrophin (PMSG) was through scientific research establishments. As the manufacture of gonadotrophins transitioned into pharmaceutical companies, the processes were required to follow certain quality standards. This concept of regulatory control of pharmaceutical manufacturing started with the FDA in 1938, which was further elaborated in 1968 to require pharmaceutical companies to demonstrate that drugs were safe and effective through controlled clinical trials.

The WHO introduced Good Manufacturing Practices (GMP) in 1968 to ensure that medicinal products were consistently produced and controlled to appropriate quality standards. The need for GMP was stimulated by several safety issues with marketed drug products in the US and the EU from as early as 1937 but the establishment of formal regulations took longer. In the US, GMP was established as a binding regulation in the US Code of Federal Regulations in 1978. In Europe, an informal GMP system (PIC, Pharmaceutical Inspection Convention) was established under the European Free Trade Association (EFTA) in 1970. On a national level, the German Medicines Act was established in 1978 but specific GMP requirements were first published in 1985, and by the European Economic Community (EEC) in 1989. GMP were also established for biological products in 1992.

The Regulatory Guidelines in Europe, and other regulatory authorities, have continued to evolve for biologics to cover all aspects of process development, analytical testing, non-clinical and clinical requirements prior to market approval.

### 2.2 Formulation development

The presentation of commercialised gonadotrophins has evolved from lyophilised hMG in ampoules, through to vials, and recently into a ready to use solution in a pen device. This HMG pen device has limited availability (mainly in Germany) and was demonstrated to be bioequivalent to powder for reconstitution (8).

Initially hMG was supplied as a 75IU dosage form, but through extensive product development, the dosage forms available now include 150IU monodose, and 300, 450 and 1050IU multidose formats.

A reusable pen injector for rFSH was first introduced in EU in 2000 by Organon, and a pre-filled pen injector in 2004 by Serono. The refined method of administration by utilizing pen injection devices was a tremendous step forward, for patient convenience and accuracy of doses that can be administered, unlike the practical challenges of dose delivery following reconstitution and administration of a lyophilised ampoule/vial urinary gonadotrophin formulation. The benefit of pen devices allowed simple and reliable self-injection at home, and the introduction of pen injectors for gonadotrophin administration has become the standard approach of all gonadotrophin manufacturers.

Following the introduction of follitropin alfa where the content of the formulation is based on the rFSH protein content (9) rather than just a reliance on the traditional *in-vivo* bioassay, it encouraged other manufacturers to consider alternative analytical methods as an alternative or perhaps a replacement of the *in-vivo* bioassay (see section below 2.3.3).

### 2.3 Analytical development

#### 2.3.1 International gonadotrophin standards

Prior to 1939 there was no uniformity in assay methods or units for gonadotrophins. The Health Organisation of the League of Nations established International Standards and defined International Units for the gonadotrophins originating from human pregnancy urine and serum. (10). The WHO Expert Committee on Biological Standardisation was established in 1947 to provide recommendations and guidelines for manufacturing, licensing and control of biotechnology products and the establishment of WHO International Reference Preparations and International Standards. The consistent assessment of FSH and LH activity in any of the commercially produced gonadotrophins requires the use of a validated and calibrated standard. The WHO Expert Committee on Biological Standardisation initiated the production and calibration of an International Reference Preparation for hMG in 1957 which was introduced in 1959 (1st IRP). A similar exercise was required for the production and calibration of an International Reference Standard for rFSH available since 1995. All the international standards rely upon a secure and representative source of gonadotrophin, and they have typically been supplied by pharmaceutical companies. International standards have also been developed for LH and hCG.

#### 2.3.2 Analytical methodology

The analytical methodology suitable to fully assess protein therapeutics, and especially gonadotrophins, was initially only applied to rFSH. This was partly due to the very high purity achieved through superior manufacturing purification processes, and the availability of analytical methodologies that could effectively be used to measure impurities, and to characterise the gonadotrophin molecule in terms of degraded forms (oxidation, deamidation, truncation) and glycosylation pattern.

Consequently, the Pharmacopeia European has issued Pharmacopeial Monographs for the active pharmaceutical ingredient (rFSH) and the final drug product (Ph.Eur., Follitropin monograph 2285, Follitropin Concentrated Solution monograph 2286). The monographs describe the minimum analytical tests which all manufacturers must use for testing recombinant FSH prior to market release. In comparison the pharmacopeia requirements for hMG are limited to standard analytical tests applicable to all aseptically manufactured products, plus the use of the *in-vivo* bioassay for FSH and LH to assess the biological potency (Menotropins USP29-NF24, BP monograph 1067).

Some manufacturers of highly purified hMG have introduced more advanced purification processes and analytical testing. Increased control of the urine donors, and the introduction of Anion Exchange Chromatography has provided a more consistent production process for the hMG and uFSH drug products from Ferring (11). An improved purification process has also been reported to produce hMG and uFSH from IBSA using Ion Exchange, Affinity and Hydrophobic Interaction Chromatographic steps (12). Although it is possible to improve the purity of any hMG product the nature and heterogeneity of the FSH glycoforms will continue to be different to the rFSH products (13). The clinical impact of these differences may be irrelevant but continues to be debated.

#### 2.3.3 Gonadotrophin Pharmacopoeia monographs

The Steelman-Pohley FSH bioassay (1953) is the only bioassay currently published in the US and EU pharmacopeias and continues to be used today to assess the FSH bioactivity in pharmaceutical products, whilst the Seminal Vesicle weight bioassay is used for LH (14).

Following the EU Convention for the Protection of Vertebrate Animals used for Experimental and other Scientific Purposes initiated in 1986 (86/609/EEC), the European Pharmacopoeia Commission intensified the review of all animal tests in pharmacopeia monographs. Directive (2010/63/EU) on the protection of animals used for scientific purposes, came into full effect in 2013 and replaced the Directive 86/609/EEC. During the drafting of the follitropin monographs, the European Directorate for the Quality of Medicines and Healthcare (EDQM) reviewed suitable alternatives to the *in-vivo* potency test. An assessment of isoelectric focusing (IEF) and capillary zone electrophoresis (CZE) as possible replacements was coordinated by the European Centre for the Validation of Alternative Methods (ECVAM) however no correlation to the potency measurement could be found, and the methods could not be universally applied to all follitropin products in Europe (15).

Some alternative physiochemical approaches have been proposed to replace the FSH *in-vivo* bioassay as a pharmaceutical analytical method to assess the FSH content (9, 16). A reporter gene assay for FSH has also been developed (17) at the National Institute for Food and Drug Control in China. More recently an *in-vitro* bioassay has been developed that is being considered as an alternative to the *in vivo* assay in the European Pharmacopeia (18). This analytical approach was approved in 2022 by the EMA to replace the FSH *in-vivo* bioassay for the testing of follitropin alfa originator and a combination of follitropin alfa/lutropin alfa. Whilst this approval signifies a substantial advancement in adopting *in-vitro* methods for FSH potency assessment, the complete integration of the *in-vitro* bioassay into the European Pharmacopoeia as a standard replacement for the *in-vivo* method has not yet occurred. It will require comprehensive validation and further consultation within the scientific and regulatory communities to ensure the new method’s robustness and reliability across various applications.

## 3 Ovarian stimulation with gonadotrophins

Following the scientific discovery of the physiological action of gonadotrophins in the period between 1925 - 1930, and that both FSH and LH are secreted from the pituitary, sources of exogenous gonadotrophins have been used to treat infertile patients (see review 19).

### 3.1 Animal and pituitary derived gonadotrophins

#### 3.1.1 Animal pituitary gonadotrophins

The ‘two-step’ ovarian stimulation protocol was first demonstrated by Mazer and Ravetz (20). They combined treatment with animal pituitary material and chorionic gonadotrophin to stimulate the human ovary. Several pharmaceutical companies started to provide commercially available animal pituitary preparations (19) until it was discovered that repeated injection of the animal extracts elicited the production of gonadotrophin antibodies which decreased the ovarian response (21). The therapeutic use of animal derived pituitary extracts demonstrated the role of the pharmaceutical industry to provide larger scale manufacturing and commercial supply.

#### 3.1.2 Pregnant mare serum gonadotrophin

In 1930, Cole & Hart (22) demonstrated that PMSG had potent gonadotrophin activity, and it was used soon after to induce ovulation (as summarised in 19). An international standard was later established in 1938, and commercially available preparations were available in Europe and the USA. However inconsistent results and adverse effects due to the foreign nature of the injected protein became apparent. Both animal pituitary extracts and PMSG were continued to be used, in conjunction with hCG extracted from human pregnancy urine, despite the antibody formation.

The FSH and LH activity of animal pituitary derived gonadotrophins and PMSG was initially assessed by a range of bioassays and standardised against a variety of national gonadotrophin standards. The lack of suitable purification technologies at that time prevented the development of a highly purified gonadotrophin and the analytical techniques did not allow any in-depth assessment of protein impurities, or only limited characterisation of the gonadotrophins present in the animal-derived materials.

#### 3.1.3 Human pituitary gonadotrophin

To avoid the problems faced with animal pituitary derived gonadotrophins, Gemzell & co-workers demonstrated that gonadotrophins could be extracted from pituitary glands of human cadavers and used to stimulate ovulation. The gonadotrophins extracted from this source were termed human pituitary gonadotrophin (HPG) or human pituitary FSH (HP-FSH) (23, 24).

A review of the clinical experience of using HPG indicated that reported studies had used different techniques to extract the gonadotrophin from the pituitary, different bioassays to assess the biological activity, and different reference standards, which had made valid comparisons very difficult. The general observation was that the incidence of multiple pregnancies was high, with a risk of ovarian hyperstimulation and other safety issues (25). A later study from Melbourne, Australia, showed that the cumulative pregnancy rate of a limited number of patients was similar to that seen in the general community (26). It was noted that multiple pregnancies and hyperstimulation could be better managed with the use of sensitive immunoassays for FSH and LH, as well as the availability of ultrasonography.

The collection and processing of post-mortem human pituitaries was largely undertaken by national human pituitary agencies in providing pituitary derived gonadotrophins. The three major national agencies included the National Hormone & Pituitary Program in the US, the Australian Human Pituitary Hormone Program and the National Health Service in the UK.

The use of pituitary derived hormones in Australia was suspended in 1985 (27) when a growth hormone recipient in the US developed Creutzfeld-Jacob Disease (CJD) (see 28). Unfortunately, in 1993, multiple cases of CJD were described and thought to be linked to the use of HPG (29). Further cases of CJD occurred in patients who had also received preparations produced by the National Pituitary Agencies in the US, Australia, New Zealand, UK, and France. The monitoring of patients treated with hormones extracted from human pituitaries continues at the National Health Agencies.

### 3.2 The development of urinary and recombinant human gonadotrophins

#### 3.2.1 Human menopausal gonadotrophin and urofollitropin

Human menopausal gonadotrophin (hMG) obtained from urine, (30), was initially introduced into clinical use in hypogonadotrophic anovulatory women using a sequential step-up/step-down regimen by Bruno Lunenfeld in 1961 (31); soon after, Lunenfeld et al. (32) reported on the use of HMG in males with gonadotrophic insufficiency. This urinary gonadotrophin eventually yielded its full potential as an ovarian stimulant following the initial work on stimulation regimens by the pioneering IVF team, Steptoe and Edwards (33).

Following the above successful reports of ovulation induction in women, the first HMG (called Pergonal^®^, from the Italian-for the gonads ‘per gonadi’) was a crude protein extract, containing about 5% gonadotrophins (see review; 34, 35). It was registered first for clinical use in Italy by Serono in 1950 and then in Israel after the pioneering work of Lunenfeld in 1963. This version of Pergonal^®^ 75, contained 75 IU of FSH and 75 IU of LH as measured by standard *in vivo* bioassays (19).

Subsequently Serono chemists developed a method to remove most of the LH activity from HMG (36) to make available an FSH only gonadotrophin (though with similar levels of impurity). This was achieved by including an additional immuno-chromatography extraction step which used an hCG polyclonal antibody, that also binds LH. This led to the clinical availability in the 1970’s, of a urinary FSH (Metrodin^®^) registered initially for use in treatment of PCOS anovulation.

In the late 1980’s some pharmaceutical companies focused all efforts into developing and promoting the use of FSH only gonadotrophins in parallel to the dramatic increase in demand for gonadotrophins fuelled by the surge in IVF treatments. Around the same time, the UK MHRA, was the first regulatory agency to approve urinary gonadotrophins for ovarian stimulation in IVF treatment cycles. Registration was based on a summary of clinical experience and no dose finding studies were conducted.

To provide clinical experience with a subcutaneous administered gonadotrophin before the launch of recombinant FSH across continents, Serono developed a process using FSH monoclonal antibodies as one of the purification steps on HMG bulk solution. This led to the first (and only) highly purified urinary FSH (urofollitropin, u-FSH HP) with a specific activity of at least 10,000 IU FSH/mg protein, containing not less than 95% FSH and with negligible LH content (37). This preparation was first commercialized in 1993 in Europe (38). Since then, there have been other companies bringing to market urinary products (both HMG and FSH) claiming to be highly purified (HP), however none of them meet the above specifications (35, 39, 40).

The development of highly purified urofollitropins marked the transition from intramuscular to subcutaneous injections which supported the self-injection of gonadotrophins at home rather than administration by a health care professional at the fertility clinic. This transition was supported by phase 1 comparative bioavailability studies showing that u-FSH and u-FSH-HP were equivalent (41).

The manufacture of more purified gonadotrophins from human menopausal urine posed significant supply issues for the various pharmaceutical companies involved. Very large volumes of urine are required, and consequently very large numbers of donors are needed to maintain a secure commercial supply. The move from HMG to urinary FSH and urofollitropin, produced using more rigorous manufacturing processes and regulatory guidelines (described below), placed even greater demands on an adequate supply of human menopausal urine. The supply challenge resulted in a phasing out of the first generation hMG’s, and product shortages during the late 1990’s.

However, during this period there were some interim attempts to address the increasing challenge of supplying hMG commercially as a 1:1 FSH: LH product. The LH activity had been reported to be preferentially lost during the manufacturing process (11). Subsequent analysis of the HMG (Menopur^®^, Ferring) demonstrated that 95% of the LH bioactivity is due to the presence of hCG (39, 40) perhaps to compensate for the loss of LH during the manufacturing process. Serono commercialized, for a short time, an HMG variant with the brand name Pergogreen^®^ that contained a 2:1 FSH: LH ratio (42). With a similar approach Organon launched Normegon^®^ (3:1 FSH: LH ratio) in some EU countries, in which no hCG was present. (43, 44).

Although HMG has proved to be a successful source of gonadotrophins for the treatment of infertility, the collection and processing of urine from multiple donors could not be considered a controlled process and any safety issues related to the quality of the urine collected from donors could not be assured. The pharmaceutical gonadotrophin products derived from human menopausal urine have been considered safe (45), but several studies have demonstrated the presence of non-gonadotrophin contaminants in HMG and urinary FSH products (35, 37, 39). A later analytical study from Van Dorsselaer et al. (46) using proteomics, did demonstrate the presence of protease-sensitive prion protein in HMG and HMG-HP pharmaceutical products. Although not seen in other markets, the UK Medicines Control Agency announced the withdrawal of Metrodin HP as a precaution in 2003, even though that risks associated with its use were ‘incalculably small’, further advising patients to switch to recombinant human FSH (rFSH), (47).

The early experiences of preparing therapeutic proteins from animal and human sourced materials has dramatically changed the regulatory requirements for the development of therapeutic proteins (EMA Guideline on the use of starting material and intermediates collected from different sources in the manufacturing of non-recombinant biological medicinal products, (48)). For urine derived hormones, exclusion criteria for selection of donors are encouraged, and manufacturers are required to estimate the potential of their manufacturing processes to reduce potential infectivity (EMA Position Statement on Creutzfeldt-Jakob Disease and Plasma-Derived and Urine-Derived Medicinal Products, first published in 2003).

In October 2022, it became apparent that issues had arose with the supply and manufacture of one marketed HMG, due to unapproved changes made by a third-party supplier in the drug substance’s manufacturing process. The company temporarily halted shipments to the UK and paused manufacturing at the supplier, leading to shortages in the UK (49). Shortages of the product are still apparent today across some EU countries and UK. However, after receiving feedback from the U.S. FDA, the company resumed shipments to the U.S. market in November 2022 (50).

International regulatory guidelines also now require the process characterisation of critical pharmaceutical drug substances (EMA Guideline on process validation for the manufacture of biotechnology-derived active substances and data to be provided in the regulatory submission, 2016), the assessment of potential immunogenicity (EMA Guideline on immunogenicity of therapeutic proteins, first published in 2007), the identification and quantification of all impurities and the qualification of all manufacturing processes and the analytical methods used to release the drug product onto the market (ICH Guideline Q8, 2006).

#### 3.2.2 Recombinant human FSH

With the exponential growth of global IVF in the 1990’s, linked to increasingly strict regulations on the industrial production of urinary gonadotrophins that limited the supply of high-quality menopausal urine, it became imperative to secure a controlled and consistent source of gonadotrophins by implementing recombinant DNA technology to produce recombinant human gonadotrophins (51), the first being rFSH, the primary gonadotrophin used in OS. **Table 2** presents the characteristics of urinary and recombinant derived gonadotropins.

**Table 2:** Characteristics of urinary and recombinant gonadotrophins.

Gonadotrophin	Purity	Mean specific FSH activity (IU/mg protein)	Injected protein per 75 IU (μg)
hMG	< 5%	~100	~750
u-FSH	< 5%	~150	370–750
hMG HP	< 70%	2000–2500	~18
u-FSH HP (urofollitropin)	> 95%	~9000	6–11
r-hFSH Follitropin beta	> 99%	10,000	8.1
Follitropin alfa	> 99%	13,636	6.1
Follitropin delta	> 99%	NA	Dosed in μg (If AMH <15pmol/l, 12mcg daily*)

The race to market a rFSH, resulted in the introduction of follitropin alpha in 1995 and follitropin beta in 1996. The clinical dossiers for registration of these two follitropins were based upon a comparison to urinary human FSH to which they had similar elimination half-lives and pharmacodynamic properties (52–55). In an opinion article Zwart-van Rijkom et al. (56) examined the diffusion of rFSH into Dutch medical practice. In this article they succinctly described the dilemma facing the Pharmaceutical Industry, purchasers and the prescriber. In the case of rFSH like many other biotech drugs, it is just an alternative option for already existing medicines obtained by extracting gonadotrophins from human menopausal urine. The introduction of rFSH demonstrated superior batch to batch consistency, was highly pure and free from urinary protein contaminants. However, what are the benefits from a clinical perspective? These latter points have been the focus of numerous trials, meta-analyses, systematic reviews and pharmaco-economic assessments for almost the last 30 years and relevant strands of the arguments will be included below. In their paper, Zwart-van Rijkom et al. (56) highlighted the main issue; although there was a perceived quality improvement in using the recombinant follitropins, there were major impacts on drug prices and levels of reimbursement in some markets that led to adoption resistance, keeping the need for cheaper (urinary) alternatives.

The first two rFSH preparations were produced using different mammalian Chinese Hamster Ovary (CHO) cell lines and methods to insert the alpha and beta human FSH subunit genes, as well as having different downstream production processes (51, 57). For these reasons, the two FSH preparations had significantly different FSH isoform profiles (follitropin beta having more basic FSH isoforms than follitropin alfa which *in vitro* have a higher affinity for the FSH receptor, resulting in higher production of cyclic adenosine monophosphate (cAMP) and other second messengers involved in the follicular response). Various erroneous claims were made based on *in-vitro* study results, and the initiation of clinical trials and subsequently symposia that promoted one being superior to the other (57–62).

The battle for market dominance of the recombinant FSH segment continued unabated for about 5 years; subsequently, through real world clinical experience of the two ‘significantly different’ FSH isoform preparations, ART specialists, academic scientists and congress organizers eventually realized that the two preparations had the same safety and efficacy profiles, including pregnancy and live birth rates (63–65).

Further gonadotrophin research and development however continued, leading to the commercialization of recombinant LH (rLH) and hCG (discussed below), as well as the use of follitropin alfa for other indications (e.g., female hypogonadotrophic hypogonadism; 66).

There were also some attempts, to develop a new generation of rFSH preparations based on selected FSH isoforms that could more closely mimic the changes observed in the normal menstrual cycle (67, 68). These FSH preparations were proposed to have a use in ovulation induction protocols to reduce the incidence of multiple follicular development. Pilot studies were conducted comparing two enriched FSH isoform pools, a more acidic (pI= 3.9-4.6) and a more basic (pI= 4.2-5.1) follitropin originating from follitropin alfa bulk material, which were separated by chromatography. (69). These were administered s.c. in a daily dose of 150 IU (acidic-8.7 mg vs basic-23.8mg FSH/vial), based on the *in vivo* rat bioassay, in down-regulated women and follicular development was compared. Administration of a pool of more acidic FSH isoforms resulted in a lower metabolic clearance, and at day 10, in the development of significantly more follicles (mean n=28) compared to the pool of basic FSH isoforms (n=16). The findings clearly demonstrated that the type of FSH isoform can have a profound effect on follicle development and supports the need for careful regulation of the FSH isoform content of pharmaceutical preparations. The results also demonstrated the limitations of the rat *in-vivo* bioassay for assigning the FSH potency of commercially available gonadotrophins and validates the use of filling by mass protein (70). However, as far as leading to potential new product opportunities, the results did not support a progression to a full development program as there was not a clear clinical benefit over what could be achieved with clinically available rFSH used at low doses in a pen device.

Organon, on the other hand, continued to invest in a long acting FSH, which was based on a patent from Irvine Boime’s group in the USA (71- discussed in next section). The company also commenced the development of a small orally bioactive LH peptide mimetic (Org 43553). This product was trialled in a human clinical study, which demonstrated ovulation (by transvaginal ultrasound and elevated serum progesterone levels) following single dose administration. However, it never reached the market (72), probably because of the clinical profile (same efficacy and presumed safety to current therapy) compared to the significant development costs that would be required. As it stands now, potential revenue from orally active gonadotrophic mimetics is low, given the huge financial investment needed to develop them and the limited cost-benefit potential in terms of efficacy and safety of an acute therapy compared to registered products. However, there are still attempts to progress the clinical development of an orally active FSH receptor agonist (discussed in Section 3.2.4).

#### 3.2.3 Long-acting recombinant human FSH

Since the approval of the first recombinant protein, insulin, in 1982 (73), many manufacturers recognised the opportunity to develop extended-release formulations, to increase the drug half-life, reduce the frequency of injections, and potentially improve patient compliance (74). Hence, there have been several attempts to develop and commercialise a modified r-FSH formulation that provided an extension of the molecule’s half-life.

In the early 2000’s, a hyperglycosylated recombinant FSH (AS900672) was developed with multiple N-linked glycosylation sites (75), which demonstrated an extended half-life in female rats. A phase II study was planned in oligo-anovulatory infertile women undergoing ovulation induction but was later abandoned.

A sustained release recombinant FSH was also developed by encapsulating rFSH into small polymeric microspheres which demonstrated a dose-dependent effect on follicular development and oestradiol production in healthy female volunteers (76). However further development was also terminated.

The approach that led to the first to market of a modified long acting FSH, was based on the work of Fares et al. (71), and involved the coupling of the carboxyl terminal peptide (CTP) of hCG to the FSH molecule (rFSH-CTP, corifollitropin alfa), which naturally extends the half-life of FSH in human serum to around 70 hours, compared with that of follitropin alfa or beta of around 36-40 hours. Because of the extended absorption and longer half-life of corifollitropin alfa, the frequency of FSH administration is reduced to once every 6 days, resulting in several injection-free days for most patients undergoing OS.

The first live birth after OS using rFSH-CTP was reported by Beckers et al. (77) and the molecule was registered in the EU for use in ART in 2 dosages in 2010 (78). However, following its commercial introduction in the EU, corifollitropin alfa did not quite live up to initial expectations, and commercialization in the USA was abandoned after multiple large US-based clinical studies had been completed.

The reasons for corifollitropin alfa’s disappointing performance within the ART therapeutic area are probably numerous, the first being that longer-acting drugs are more suited for chronic disease conditions. A course of OS typically takes a median of 11 days. The impact of reducing the number of injections during an OS cycle is limited, as other injections (e.g., GnRH analogue injections) are still necessary. Additionally, as an injection of corifollitropin alfa was developed to sufficiently elevate FSH for approximately 7 days, supplementary daily FSH injections maybe required thereafter in some patients if the appropriate follicular response has not yet been achieved. This leads to an FSH naïve patient having to potentially learn two FSH injection techniques during the same treatment cycle.

However, in 2022, corifollitropin alfa combined with hCG was registered by EMA to treat adolescent boys aged 14 years and older who have delayed or absent puberty due to hypogonadotropic hypogonadism. Here FSH is given for around 64 weeks to increase testicular volume, thus supporting the medical need for a long-acting FSH to reduce injection frequency and improve compliance.

Alternative fusion protein candidates have also been reported. An FSH Fc fusion protein was developed by fusing an Fc/Fc heterodimer to the beta chain of FSH (79). An extended half-life was seen in both rats and cynomolgus monkeys. It is reported that the fusion protein is currently in phase III trials in China. Another FSH Fusion protein was developed (80) using genetic engineering to create an FSH-IgG4 Fc fusion protein. The fusion protein demonstrated a longer half-life in Sprague-Dawley rats when compared to follitropin alfa. Phase 1 clinical trials are planned to examine the pharmacokinetic and pharmacodynamic properties of this molecule.

The development of another long-acting r-FSH has been recently published (81), which has been proposed as a potentially important treatment of patients with hypogonadotropic hypogonadism. However, with the registration of corifollitropin alfa in such an indication and considering the rarity of the condition it would seem unlikely that this product will advance to a further stage of development.

#### 3.2.4 Additional development approaches

Although oral administration of therapeutic proteins is usually disregarded due to low bioavailability, a study of orally administered rFSH in a PCOS rat model (82), demonstrated some attenuation of the characteristics associated with PCOS, however, the mechanism of action was not apparent.

In another development, researchers at Syntonix developed an FSH-Fc fusion protein by linking the intact FSH molecule, or the alpha and beta subunits of FSH, with the Fc domain of immunoglobulin G1. The two fusion proteins were used to examine carrier-mediated transport via the Fc receptor across intestinal epithelia in neonatal rats, and lung epithelia in cynomolgus monkeys (83). The FSH-Fc proteins demonstrated increased blood stability and improved bioactivity. The authors proposed that the FSH-Fc fusion proteins may offer the potential for oral or pulmonary delivery of FSH.

The research efforts related to finding a small molecule able to induce ovarian stimulation via the FSH receptor have been presented in a review (84) and individual research efforts on developing specific FSH receptor agonists have been published (85–88), but despite the numerous publications few have been actively pursued into clinical development. However, Cellmatix have announced in 2024 that they have nominated a lead compound in its world’s first oral FSH receptor agonist drug development program (www.celmatix.com Press Release, 2024).

Another novel approach being developed by IGYXOS, involves the use of a monoclonal antibody that binds to both the α and β subunits of FSH, stabilizing the dimer and enhancing FSH receptor interaction. This increased FSH activation could potentially treat infertility in men and women more effectively and simplify treatment regimens, either alone or with standard gonadotrophin treatments (89, 90). Currently this humanised monoclonal antibody is entering Phase I in healthy male and female volunteers (90, 91).

#### 3.2.5 Alternative dosing regimens and administration sites

As with other therapeutic proteins administered primarily by intra-muscular or subcutaneous routes, alternative methods of administration have been proposed for gonadotrophins.

Following numerous studies going back to the 1980’s with alternative day, every third day injections of gonadotrophins (92–94), an intermittent vaginal injection approach has also been reported (95–97) with outcomes not inferior to daily conventional abdominal administration. The studies suggest faster absorption, slower elimination, and a larger systemic exposure via the intermittent vaginal route. However, it should be noted that vaginal injection is not conducive to self-administration by the patient.

The same group has also investigated abdominal mesotherapy injection which provided a prolonged elevation of serum FSH and could reduce the number of rFSH injections or lower the rFSH dose needed (98).

#### 3.2.6 Recombinant human LH and hCG

Recombinant LH (rLH) has met some challenges in demonstrating benefit in women undergoing ovarian stimulation for IVF/ICSI and in 2000, rLH was registered by EMA in combination with rFSH, for ovarian stimulation in women with severe gonadotrophin deficiency such as hypogonadal hypogonadism (99, 100).

The 1998 study suggested that increasing exposure to LH during the follicular phase reduces the number of growing follicles, providing clinical evidence for the ‘LH ceiling effect’, (101–103).

Further studies supported that 75 IU of LH activity/day is sufficient for normal follicular development and luteal phase function in hypogonadal hypogonadism (HH) (104). Subsequent studies confirmed these findings using 75 IU rLH/day in combination with 150 IU rFSH in HH patients (105, 106). Eventually, a lyophilized combination of follitropin alfa/lutropin alfa (150IU FSH:75IU LH) was launched in the EU in 2007 for this indication (see next section 3.2.7).

The clinical utility of the LH ceiling effect was further explored in a series of studies. Subsequent findings from a pilot study demonstrated that high doses of rLH in the late follicular phase suppressed follicular development both in HH as well as WHO II anovulatory women (100). This observation was similar to that reported by Filicori (107) when utilising hCG-supplemented HMG.

Hugues et al. (108) further investigated if rLH could be used to achieve mono ovulation for conception *in vivo*. In this elegant placebo-controlled, double-blind study, four doses of rLH (150, 300, 660, 1325 IU) were given daily in the late follicular phase (in combination with a fixed dose of 37.5IU FSH) to find the optimal dose that could maintain growth of a dominant follicle, whilst leading to atresia of secondary ones. The study was conducted on WHO II anovulatory women who were experiencing an excessive ovarian response to FSH treatment. The results demonstrated that doses up to 660 IU rLH/day increased the proportion of patients developing a single dominant follicle compared to placebo. However, despite these promising findings, clinical development was not continued, again mainly due to the projected development costs for a limited clinical indication.

Additionally, in a randomised, double-blind, double-placebo study, the use of rLH (5,000, 15,000, 30,000, or 15,000 + 10,000 IU with the second injection administered 3 days after the first) was also explored as a substitute for a 5000 IU hCG injection, to trigger follicular and oocyte maturation (109). rLH was well tolerated and no moderate or severe OHSS was reported following a single dose of 30,000 IU. There was however a consistent non-significant trend toward lower numbers of oocytes, clinical pregnancies and live birth rates in patients treated with lower doses of rLH. The project was halted due to manufacturing constraints as a large dose of rLH and possibly two injections, would be needed to obtain the most optimal results (between 15’000 – 30’000 IU rLH) compared to the established 5000IU uhCG trigger dose. Strong pharmaco-economic arguments would also be needed.

In contrast, recombinant hCG (rhCG) registration was simpler, as a follow-on substitute for urinary hCG used as the final trigger for final oocyte maturation in infertile women undergoing IVF/ICSI (110, 111).

#### 3.2.7 rLH/rFSH combination for assisted reproductive techniques

In the late 1990’s a development plan was proposed for a recombinant combination product which could compete head-to-head with HMG and any future threat of FSH biosimilars. This led to the commercialisation in women with severe gonadotrophin deficiency (hypogonadotrophic hypogonadism), of a product with a 2:1 FSH: LH preparation – in effect a recombinant ‘Pergogreen’. A small dose-finding study performed in women aged 38-42 years undergoing IVF suggested that a fixed ratio of 2:1 or 3:1 FSH: LH was beneficial in terms of the number of oocytes retrieved and pregnancy rates (112).

It wasn’t until 2012 that clinical development for an ART indication was initiated for rLH. At this time a Phase IIIb trial was initiated, to examine the utility of LH supplementation (2FSH:1LH ratio) vs FSH alone, in the late follicular phase of IVF patients aged 36-40 years (113). The primary objective was equivalence in number of oocytes retrieved per patient. However, less oocytes were retrieved with LH supplementation and therefore the study did not reach the predefined limit of equivalence. Thus, again this study supported the LH ceiling hypothesis and previous findings from ovulation induction of also a modulatory role for LH on the follicle cohort in ART.

Around the same time, a meta-analysis (114), in women with a poor ovarian response to stimulation suggested that rLH supplementation resulted in a reverse finding- an increase in the number of oocytes retrieved. However, that meta-analysis also included what are defined as Poseidon Group 1 and 2 patients (115, 116). These were patients with an adequate ovarian reserve who unexpectedly exhibited a poor ovarian response to stimulation with FSH-only preparations, but who were reported to respond adequately if supplemented with rLH.

On the other hand, a real-world evidence observational study on over 9000 low prognosis patients classified according to the POSEIDON criteria (117) did not demonstrate a benefit of LH activity supplementation on outcomes. A logistic regression analysis revealed that the POSEIDON grouping, number of embryos obtained, number of ET cycles per patient, number of oocytes collected, female age, duration of infertility and BMI were relevant predictors for Cumulative Delivery Rate (CDR; P < 0.001). Gonadotrophin type, total gonadotrophin dose, type of GnRH analogue and ovulation trigger were all not significantly associated with CDR.

Even though the findings of the Lehert et al. (114) meta-analysis were not aligned with previous research demonstrating LH’s impact on follicle atresia, it prompted the initiation of a large Phase IIIa IVF trial to compare FSH/LH in a 2.1 ratio to FSH-only in women with poor ovarian reserve, aligned with the Bologna ESHRE criteria. However, despite being powered to show a difference of one oocyte in favour of the FSH/LH group, the study failed to demonstrate this (118), as well as no improvement in live birth rates. The Bologna POR classification is widely acknowledged to encompass a heterogeneous population. The patients included in the study can be retrospectively divided into three basic severity scores, known as PROsPer score (mild, moderate, and severe), based on age, ovarian reserve, and the number of oocytes retrieved during a previous stimulation (119). Patients with a moderate and severe score had a higher live birth rate and a lower pregnancy loss rate when treated with FSH/LH compared to FSH-only, according to this retrospective analysis.

Another study, which also used the same system and included 9667 Bologna criteria POR cycles, reported that moderate and severe PROsPer groups had a significantly higher cumulative live birth rate when treated with FSH/LH as compared to FSH-only (120). It was concluded that FSH/LH treatment is more effective than FSH-only treatment for patients with moderate and severe scores on the PROsPeR system. However, the role of LH in moderate and severe PROsPer groups remains hypothetical until appropriate randomised clinical trials are completed.

Conforti et al. (121) conducted a meta-analysis which found that although fewer oocytes were retrieved in women aged 35 or older who received rFSH/r-hLH compared to rFSH alone, the clinical pregnancy rate was higher in the rLH supplemented groups. In a very recent analysis of the German IVF register, it was confirmed that patients 35-40 years who received r-FSH/r-LH treatment instead of r-FSH had significantly less oocytes (-1.74 (95% CI -2.00 to -1.48) but with no difference in the live birth (absolute difference +2.3% (95% CI: 0.2-3.9) or cumulative live birth rate (absolute difference -1.2% (95% CI: -3.4-0.9) (122).

In physiology, LH plays a dominant role from the mid to late follicular phase (123). Studies on rodent models have consistently shown that FSH inhibits granulosa cell apoptosis and follicular atresia in a dose-response manner (124), while also promoting granulosa cell proliferation (125). In contrast, LH/hCG have been found to be less effective in suppressing apoptosis (126, 127). These *in vitro* findings were encapsulated in the LH ceiling hypothesis (102) and demonstrated in a clinical model described earlier, where administration of rLH through the period of FSH stimulation as well as in the mid to late follicular phase led to suppression of follicle development (100, 113). This development related ‘LH ceiling’, when breached resulted in several effects such as complete follicular growth arrest to impaired ability of a follicle to luteinize. Thus, there is a clear modulatory role on follicular development.

Apart from the above-mentioned retrospective studies and meta-analyses (128), an RCT in patients stimulated with different FSH starting doses, but similar total IU’s of FSH+LH activity, demonstrated no differences in ongoing clinical pregnancy rates in women aged 36-39 years and in those <36 years (129). More recently other small studies failed to find any difference (130, 131). Thus, the LH supplementation saga continues and the cohort of patients who may benefit is still elusive. This reflects a typical conundrum for pharmaceutical companies: a developed drug looking for a wider indication.

#### 3.2.8 FSH biosimilars

Following the introduction of EMA guidelines on biosimilar development in 2004 and specific rFSH biosimilar guidelines in 2013, new players have entered the field with the introduction of biosimilar follitropin alfa preparations.

In a commentary, de Mora and Fauser (132) described the background of a biosimilar registration. According to the regulatory definition, a biosimilar is a biological medicine that has been demonstrated, through a series of physico-chemical, *in-vitro*, *in-vivo* tests, and confirmatory Phase I and Phase III studies to be similar/equivalent in quality, safety, and efficacy to the reference medicinal product. However, this definition is only valid if registration is by one of the Strict Regulatory Agencies (now termed WHO- Listed Authorities), including those of US FDA, Japan, Canada, Australia, European Economic Area (EEA) and UK MHRA (133). It is incorrect therefore, to assign the term biosimilar (as defined and regulated by the WHO-Listed Authorities) to a rFSH product developed and trialled only for example in China (134). Since the launch of the first biosimilar in 2006, the EMA has approved a total of more than 90 biosimilars and evidence acquired to date suggests that these biosimilars can be used as safely and effectively in their approved indications as other biological medicines (135). Additionally, the EMA & Heads of Medicines Agencies (HMA), have recently issued a statement confirming that biosimilar medicines approved in the EU are interchangeable with their reference medicine or with an equivalent biosimilar (136).

Despite the rigorous scientific basis for registration of biosimilars adopted by the WHO- Listed Authorities, there is some ongoing confusion within the reproductive medicine community on what actually is a biosimilar, leading to resistance in their use. The suggested differences reported in early meta-analyses and associated reviews between biosimilars, and the originator seem to revolve around subtle variations in the FSH isoform (glycan) composition, (137, 138). These are minor compared to those between follitropin alfa and beta (62, 139) and urinary gonadotrophins (40). A retrospective study (data from 2013-2018 that contained only 5% of treatments utilising a FSH biosimilar) using the French payments database (SNDS) suggested that biosimilar rFSH (and also HMG) were not as effective as the originator follitropin alfa (140). There are, however, numerous issues with this database, notably there is no information on important biological parameters such as ovarian reserve markers or oocytes retrieved. However, other data from a large European multicentre comparative post approval trial required by the EMA (141), comparing a biosimilar to originator follitropin alfa, demonstrated that the OHSS incidence proportion and severity, as well as pregnancy and live birth rates were similar. Overall, real world evidence published to date, including another French multicentre analysis of over 6500 treatment cycles with a follitropin alfa biosimilar (142) and an analysis of over 7000 oocyte donor– recipient cycles (143) using FSH biosimilars versus the originator, confirmed that the 2 biosimilar FSH preparations available in the EEA, UK and Australia are the same quality replacements for the originator follitropin alfa (144).

Unfortunately, there is still within the ART community, a repetitive misunderstanding of the core scientific principles laid down by the EMA of biosimilarity (145). In reply, de Mora and Howles (133) clearly described in the words of EMA experts, the foundation of demonstrating biosimilarity ‘*it is undisputed that a comprehensive analytical and functional comparison of the biosimilar candidate and the reference product are the mainstay for establishing biosimilarity’.* The argument put forward by Venetis and Mol (145) that RCT’s are the best way to evaluate effectiveness for a biosimilar is erroneous as it would be the least sensitive way to pick up differences. These two co-authors have again been involved in recently published systematic review and meta-analysis (146) comparing ‘biosimilars’ vs the originator follitropin alfa. The fundamental flaw of this meta-analysis again lies in incorporating four rFSH products that have not been registered by a WHO-Listed Authority, and thus biosimilarity is not certain. Additionally, another rFSH product included was not registered as a biosimilar. Of the two EU approved biosimilars, pooling the respective data did not reveal a significant difference in live birth rates. The non-biosimilarity of two of the FSH products incorporated into the above meta-analysis has been clearly demonstrated by Manzi et al. (147). They identified differences in N-glycosylation occupancy, antennarity, sialylation and oxidation between the reference follitropin alfa and the other FSH preparations analysed, none of the latter having been registered by any of the WHO-Listed Authorities.

#### 3.2.9 Follitropin delta and epsilon

Within the last 10 years, a new rFSH (follitropin delta) with different pharmacokinetic properties compared to the existing rFSH and u-FSH products was launched. To produce follitropin delta, a different cell line (PER.C6 of human foetal retinal origin) was used to those for follitropin alfa and delta, and due to its higher proportion of complex carbohydrate structures and hence higher overall sialic acid content, it has a significantly longer elimination half-life (30h vs 24h for follitropin alfa, 148; the EPAR summary for the public, Rekovelle follitropin delta 2017). Consequently, the product is dosed according to an algorithm in µg based on a recent determination (within the last 12 months) of serum Anti-Mullerian hormone (AMH) concentration, (measured by one of 3 specific assay systems) and weight (kg) of the IVF patient (Rekovelle summary of product characteristics).

Follitropin delta, dosed according to the algorithm, aims to avoid extremes in ovarian response (149). The conclusion from the Committee for Medicinal Products for Human Use (CHMP) was that follitropin delta was as effective as follitropin alfa at stimulating the ovaries and that its “safety profile was considered acceptable and like that of the comparator, follitropin alfa” (150). However, due to the significant longitudinal and intra-cycle variations in AMH levels, independent of age, concerns have been raised that a single AMH measurement may be insufficient for determining the FSH dose (151). Additionally, the lack of an international standard for AMH limits comparison between AMH assays (152) and whilst a purified human AMH preparation (code 16/190) was evaluated by the WHO, it showed inconsistent commutability across different commercial assays (153).

One of the major criticisms of the clinical data published for follitropin delta is that the product was compared against the comparator, follitropin alfa, administered at a fixed starting dose and adjustable on day 5, irrespective of age or ovarian reserve. Another trial using the same approach (154), as well as an individual meta-analysis (155), has perpetrated the message that follitropin delta reduces safety risks and is more effective regarding gonadotrophin dosage vs conventional dosing. However, this is not routine clinical practice (156).

Other companies (Glycotope GmbH, Berlin Germany) have been active in the development of yet another injectable FSH (FSH-GEX^®^; follitropin epsilon) which also has different pharmacodynamic properties from follitropin alfa. Follitropin epsilon has undergone Phase I, and II trials (157, 158).

Following the experience to date with follitropin delta, the question could be raised whether entry of yet another follitropin with different pharmacokinetics compared to tried and tested follitropin alpha and beta, as well as urinary FSH, will yield any important clinical advantages.

## 4 Use of gonadotrophins in ovarian stimulation protocols

This section will review and discuss how perceived differences have developed between FSH alone and LH/hCG combinations when combined with a GnRH agonist (GnRHa) and the subsequent impact of the introduction of GnRH antagonists into OS protocols.

### 4.1 Which gonadotrophin to use in the long GnRHa down-regulation protocol?

The use of GnRHa preparations in combination with gonadotrophins for fertility treatment were first proposed in the early 80’s (159, 160) and specifically in IVF by Porter et al. (161). At that time, the most common stimulation protocols used a combination of clomiphene citrate and HMG or HMG alone. However, there were multiple problems associated with their use, in particular the deleterious effects of a premature LH surge prior to hCG triggered follicular maturation, oocyte maturity and luteal phase advancement (162).

These GnRHa products were quickly incorporated into OS protocols (163) and first approved for pituitary down regulation in IVF in the late ‘80’s early ‘90’s. The “long GnRHa down-regulation” protocol which involves a pre- treatment phase of typically 6-10 days before starting ovarian stimulation, swiftly became the gold standard for ovarian stimulation in IVF, in spite the higher total dose of FSH required compared to the existing regimens. This rapid swing in practice was because a virtually eliminated cycle cancellation due to spontaneous ovulation, leading to significantly improved success rates and facilitating scheduling of the IVF treatment. However, it has been clear from early studies that the recommended daily dose of GnRHa, utilized in an ovarian stimulation protocol was generally higher than the minimal effective dose (164). Additionally, due to the different amino acid substitutions used by manufacturers at position 6 and 10, the potency of different agonists varies, as well as due to the formulation, daily (injection or intranasal) vs. depot administration (165). This complicates the comparison of results obtained across studies using different GnRHa compounds, moreover, these product characteristics certainly impacted the outcome of stimulation when comparing FSH only to FSH/LH (HMG) preparations (166) as aptly illustrated in women with complete gonadotrophic deficiency (167).

With the long GnRHa down-regulation protocol well established, the need for LH activity supplementation during ovarian stimulation was questioned and re-explored. Urinary FSH preparations were reported to be equal, or even better, than HMG products (168–171).

On the other hand, a wide range of Phase IV clinical trials emerged suggesting another paradigm: “when coupled to uFSH, hCG driven LH activity is actually more effective than rFSH”. This theory was ably supported by clinical trials that used a potent GnRHa and a fixed FSH dosing regimen (The European and Israeli Study Group on highly purified hMG versus rFSH 2002; 172). Concerns about the merit of these studies were described and dissected by Trew (173), who concluded that ‘‘some of the pharmacodynamic differences alluded to in this trial (MERIT) may be protocol-driven rather than LH-activity derived’’. However, all such concerns were buried by the clinical community who wanted a wider choice of gonadotrophins, particularly one with an LH containing alternative for ovarian stimulation, which in some markets was less expensive than rFSH or rFSH/rLH.

In fact, a potential protocol driven effect was apparent in the Cochrane systematic review including 42 trials on recombinant versus urinary gonadotrophins for ovarian stimulation. Within this analysis the authors noted that the live birth rates were borderline lower for rFSH vs urinary gonadotrophins in the sponsored trials (6 trials, N=2817; OR 0.84, 95% CI 0.71-1.00). This was probably influenced by the type of GnRHa used in the long protocol and the fixed dose FSH regimen. However, the overall conclusion demonstrated that if calculating the fresh transfer cycle only, and not exploring the cumulative live birth rate (LBR)- there was no differences in clinical pregnancy, ovarian hyperstimulation syndrome (OHSS), or live birth when rFSH was compared to urinary gonadotrophins within any of the down regulation groups (7). The ESHRE Guideline on ovarian stimulation for IVF/ICSI (2019), also equally recommended the use of rFSH or HMG.

There has recently been an updated Cochrane systematic review and network meta-analysis providing the most comprehensive summary of ovarian stimulation protocols for ART (174). In summary, they reported an uncertainty of a difference between gonadotrophins in long GnRH agonist protocols for LBR and OHSS. GnRH antagonist with HMG (vs rFSH) probably reduces OHSS in high responders and in normal/high responding patients, and in line with the LH ceiling hypothesis, LH may reduce oocyte number, but effect on frozen embryo number is yet uncertain.

The hCG driven, LH activity ‘story’, in several publications has suggested a positive association with mid-follicular concentrations of exogenous hCG and live birth rates in women co-treated with a long GnRHa protocol (175). This long-standing “mantra” delivered in publications and at congresses for more than 15 years, is now under scrutiny following publication of a pharmaceutical sponsored dose finding trial (176) using r-hCG (choriogonadotropin beta expressed in a human cell line). In this well designed (placebo controlled, double blind) randomised clinical trial, a range of choriogonadotropin beta doses (0,1,2,4,8,12 ug) were combined with individualized follitropin delta doses for ovarian stimulation in a long GnRH agonist protocol. The primary endpoint was the number of good quality blastocysts. Not surprisingly if we consider physiology, fewer oocytes were observed in all groups receiving r-hCG. Additionally, and maybe more concerning, less good quality blastocysts were obtained and the ongoing pregnancy rate per started cycle was 43% in the follitropin delta comparator, but lower (33% on average) across all choriogonadotropin beta dosing groups.

Whilst the impact of choriogonadotropin beta on follicle and oocyte number are similar to that reported previously using HMG, the additional observations from this placebo controlled double blind trial demonstrating generally less good quality blastocysts, raises further questions to those discussed above on the benefit of supplemental LH-like activity during ovarian stimulation in the general IVF population (see review by 177).

### 4.2 The switch to GnRH antagonists in ovarian stimulation protocols

The first GnRH antagonist, cetrorelix was introduced to the market in 1999 followed a year later by ganirelix. It took a rather prolonged gestation (almost 10 years) before this group of GnRH analogues became established in IVF protocols. This was despite there being important advantages for patients of using GnRH antagonists in terms of less injections, overall shorter treatment duration, lower FSH consumption, significantly lower risk of OHSS (178) and the possibility to trigger final oocyte maturation effectively with a bolus of GnRH agonist (179, 180). Generally, less oocytes were reported from the early studies to be collected vs GnRH agonist protocol and some of the early supporting clinical data suggested lower pregnancy rates, as well as there being some concern as to the most appropriate daily dose (181). This concern of lower ongoing pregnancy rates in the general IVF population continued up until 2017 (182) as reported in a meta-analysis comprising 50 studies. However, in the most recent systematic review and network analysis (174), the evidence demonstrated that in women with predicted normal or high response, the use of GnRH antagonist protocols may result in little to no difference in live births and a reduction in OHSS vs long GnRH agonist protocol. Additionally, in predicted poor responders, the overall evidence suggests no differences in terms of safety or efficacy between the GnRH agonist and antagonist protocols (183). It seems therefore that the clinical community has now learnt how best to use the GnRH antagonist protocol.

## 5 Discussion and future recommendations

The Pharmaceutical Industry has significantly shaped the way we practice ovarian stimulation in ovulation induction and ART (see summary **Table 3**).

**Table 3:** Summary Impact of Pharmaceutical Industry on Fertility therapeutic area.

Development Activity	Outcome
Gonadotrophin Supply	Since the early 1970’s, increasingly secure supply, improved purity
HMG Quality	Improved with evolving regulatory guidelines
Recombinant DNA (rDNA) Technology	Introduction of originator rFSH, rLH, rhCG drug products and subsequently FSH biosimilars
Administration Formulations	More patient friendly (liquid) and pen devices
Long Acting FSH Preparations	Harnessing rDNA technology has provided the framework to develop novel FSH molecules with longer half-life.
GnRH Antagonists	Introduction of shorter, flexible OS protocols, lower OHSS risk
Training and Education	Advance medical knowledge and best practices

The immense pressure on drug development often results in practical considerations that take the path of least resistance to market entry and to maximise returns for future research and development. As discussed in the introduction and illustrated in our medical field (**Table 1**), development of a gonadotrophin drug (e.g. long acting FSH) or a GnRH analogue is NOT a rapid process and may result in commercialisation after therapeutic protocols have advanced, leaving the newly registered product seemingly outdated.

There have been several brave attempts to bring new molecules/indications to our field (e.g., development phase: lutropin alfa as follicular trigger or to suppress secondary follicle development, an LH small molecule mimetic for oral administration) but they did not continue to be developed for possible regulatory submission.

There are still insufficient randomised controlled trials (RCT) that are double blind and placebo-controlled to truly address unanswered questions. Open, even ‘assessor blind’ trials, suffer from a high risk of bias. Until then we will continue to be often misguided by erroneous meta-analyses. An important element that is not always considered when analysing a trial design, compiling a meta-analysis or systematic review is to ask the question, ‘how much of the result is influenced/explained by basic physiology’. For example, the type of GnRH agonist used in a comparative HMG vs FSH only trial can influence the total gonadotrophin dose, oestradiol output, days of stimulation (184) as well as late follicular progesterone levels and pregnancy rates (166, 172). Additionally, the 114 meta-analysis demonstrated that LH supplementation increased the number of oocytes retrieved in ART cycles.

The Pharmaceutical industry is the only player today that can consider the cost of any statistically powered (for registration purposes 90%), double-blind RCT, and ultimately, it is in their interest to design rigorous studies to demonstrate the clinical value of their drug product following the high cost of development. Inevitably such an RCT will have strict inclusion and exclusion criteria to limit the patient variables which will not necessarily reflect the patient demographics of a typical IVF clinic. However, it is vitally important in a complex treatment process such as ART to have a well-defined patient population as a basis for any RCT.

Whilst there are now excellent initiatives to develop a core outcome set of variables for future fertility research, (185), consensus documents on the development of ART laboratory performance indicators (186), performance indicators for clinical practice in ART (187) and both clinical and laboratory key performance indicators (188), there is a need to translate and apply these recommendations into clinical trial protocols. It is to this point that reproductive specialists, associated societies and the pharmaceutical industry work to develop clear, unambiguous guidelines on study design, inclusion and exclusion criteria, incorporation of key treatment performance indicators, and stricter control of pivotal variables (e.g., embryo development and assessment as well as embryo transfer procedure), including a standardized luteal phase support regimen, (a research area that has recently been under intense scrutiny, see 189, 190). Such guidelines would better ensure truly balanced randomised clinical trials where ongoing clinical pregnancy rate is the primary endpoint.

When considering ovarian stimulation and the use of different commercialised FSH gonadotrophins within a complex treatment process such as ART, we advocate a cessation of comparative studies unless there is a focus on answering core questions that may lead to improved treatment outcomes. There is a renewed need for the Pharmaceutical Industry to collaborate with the clinical community to address the unanswered questions such as ‘does LH activity supplementation’ deliver unequivocally better outcomes, especially in women of advanced maternal age. The medical community have been involved in trying to answer this for more than 30 years and we do not seem to be any closer to an answer, especially with the increased acceptance and use of GnRH antagonist protocols (see review 177).

Gonadotrophins from either human urine or recombinant technology have adequately demonstrated that ovarian stimulation can be effective and safe, leading to successful pregnancies and live births in most categories of female and male infertility. Today, the evidence clearly demonstrates from single IVF units, multicentre analyses or national databases, that the number of oocytes retrieved from OS is a critical predictor of a patient’s chance (linked to their age) to pregnancy and the cumulative live birth rate (191–195). Hence, the pivotal role that personalised FSH dosing, based on ovarian biomarkers such as AMH, plays in an ART treatment. Over the last 15 years, further significant efforts have been directed at assessing the quality of the embryo for implantation in IVF (e.g. preimplantation genetic testing for aneuploidy, time lapse imaging, use of AI in aiding the discrimination of embryo viability). The use of machine learning models to assist in decision making for the FSH starting dosing and time of follicular trigger, may also contribute to optimizing the number of mature oocytes retrieved and hence ART outcomes (196–198). The above tools should also be considered for supporting the validity of any further randomised studies comparing drug regimens or other technologies introduced into the ART treatment process.

One major challenge remains largely unsolved, and that is to improve the implantation of a viable embryo, resulting eventually in a live birth. There are also numerous, non-gonadotrophin pathways for the pharmaceutical industry to investigate potential therapeutic options to improve oocyte competence, embryo viability and assist implantation. New technologies on the far horizon may well dramatically reduce/replace injectable gonadotrophin use, e.g., *in-vitro* follicular maturation and growth (199), the reprogramming of somatic cells to produce gametes (200) and new *in-vitro* maturation techniques (201). However, as of today, gonadotrophins are well and firmly established as a cornerstone of successful fertility treatment.
